# Triarchic Psychopathy Dimensions in Chimpanzees (*Pan troglodytes*): Investigating Associations with Genetic Variation in the Vasopressin Receptor 1A Gene

**DOI:** 10.3389/fnins.2017.00407

**Published:** 2017-07-17

**Authors:** Robert D. Latzman, Steven J. Schapiro, William D. Hopkins

**Affiliations:** ^1^Department of Psychology, Georgia State University Atlanta, GA, United States; ^2^Michael E. Keeling Center for Comparative Medicine and Research, University of Texas MD Anderson Cancer Center Bastrop, TX, United States; ^3^Department of Experimental Medicine, University of Copenhagen Copenhagen, Denmark; ^4^Neuroscience Institute, Georgia State University Atlanta, GA, United States; ^5^Division of Developmental and Cognitive Neurosciences, Yerkes National Primate Research Center Atlanta, GA, United States

**Keywords:** vasopressin, AVPR1A, psychopathy, chimpanzees, nonhuman primate models

## Abstract

Vasopressin is a neuropeptide known to be associated with the development and evolution of complex socio-emotional behaviors including those relevant to psychopathic personality. In both humans and chimpanzees, recent research suggests a strong genetic contribution to individual variation in psychopathic traits. To date, however, little is known concerning specific genes that might explain the observed heritability of psychopathy. In a relatively large sample of captive chimpanzees (*N* = 164), the current study thus sought to investigate gene-environment associations between triarchic psychopathy dimensions (i.e., disinhibition, meanness, and boldness) and (1) early social rearing experiences and (2) polymorphisms in the promoter region of the V1A receptor gene (AVPR1A). Among chimpanzees raised by their biological conspecific mothers, AVPR1A was found to uniquely explain variability in disinhibition and in sex-specific ways for boldness and a total psychopathy score; however, in contrast, no significant associations were found between AVPR1A and any of the triarchic psychopathy dimensions in chimpanzees raised the first 3 years of life in a human nursery. Thus, when considered in its entirety, results suggest an important contributory influence of V1A receptor genotype variation in the explanation of the development of psychopathy under some but not all early rearing conditions. Results of the current study provide additional support for the assertion that psychopathic tendencies are rooted in basic, evolutionarily-meaningful dispositions, and provide support for a primate-translational operationalization of key neurobehavioral constructs relevant both to psychopathy and to broader forms of psychopathology.

## Introduction

Psychopathic personality (psychopathy) is a condition that involves severe disturbance in behavioral control, social relations, and emotional experiences concealed by an outward appearance of normalcy. Although historically studied predominantly in adult forensic samples, it has become clear that psychopathy is a multi-faceted condition that includes tendencies grounded in basic biobehavioral dispositions that vary continuously within the human population (Patrick et al., 2009; Lilienfeld et al., 2015). That is, individuals vary on psychopathic traits in degree, rather than kind. Viewed in this way, understanding of psychopathy can be advanced through study of psychopathy-related trait dimensions in a range of populations, including both clinical and non-clinical samples (Lilienfeld, 1994; Hall and Benning, 2006; Salekin, 2006). Consistent with this conceptualization, recent work has sought to more accurately capture the dimensions of the construct, through the explication of its component dispositional trait dimensions (e.g., Patrick et al., 2009; Marcus et al., 2011; Poythress and Hall, 2011). Developed for this purpose, the triarchic model (Patrick et al., 2009) characterizes psychopathy as a configuration of three dimensional traits explicitly linked to underlying biological systems: *boldness, meanness*, and *disinhibition*.

Within this framework, investigations of these biobehavioral dimensions have been extended to our closest living relatives, chimpanzees (Latzman et al., 2016a), providing a basis for comparative research on the evolutionary and neurobiological foundations of psychopathy. Similar to in humans (Farrington, 2006; Larsson et al., 2006; Tuvblad et al., 2016), recent quantitative genetics work in chimpanzees suggests that variability in psychopathy dimensions is heritable (Latzman et al., 2017). To date, however, little is known concerning specific genes that might explain the heritability of psychopathy. In a relatively large sample of chimpanzees, the current study thus aimed to examine one particularly promising gene, AVPR1A, a gene that underlies arginine-vasopressin (AVP), a neuropeptide known to associate with a range of psychopathy-relevant social behaviors.

The now influential triarchic model of psychopathy (Patrick et al., 2009; Patrick and Drislane, 2015) characterizes the symptomatic components of psychopathy in terms of three biobehavioral trait constructs (i.e., traits with clear referents in biology and behavior): *disinhibition, meanness, and boldness*. Disinhibition reflects an externalizing liability and phenotypic propensity toward impulse control problems. These problems include a lack of planfulness and foresight, difficulties regulating affect and urges and delaying immediate gratification, and deficient behavioral restraint. Meanness corresponds to the callous aggression subdimension of the externalizing spectrum of psychopathology and includes deficient empathy, disdain for and lack of close relationships, exploitativeness, rebelliousness, excitement seeking, and empowerment through cruelty (Krueger et al., 2007). Lastly, boldness encompasses low levels of fear/avoidance (Kramer et al., 2012), expressed as a capacity to remain calm in situations involving threat, an ability to recover quickly from stressful events, high self-assurance and social efficacy, and an easiness with unfamiliarity and danger (Lilienfeld et al., 2012, in press).

As noted above, Latzman et al. (2016a) developed a chimpanzee operationalization of psychopathic personality organized around the triarchic model. Specifically, drawing on caretaker-rated items from an existing primate personality instrument, Latzman et al. used a consensus rating approach to formulate scale measures of the three triarchic model constructs for use with chimpanzees. These Chimpanzee Triarchic (CHMP-Tri) scales were then validated both in terms of their associations with performance on behavioral tasks and their translational relevance to humans. As noted by Latzman et al. (2016a), it is important to note that this model was not developed to derive some ways of characterizing some chimpanzees as “psychopaths” in a clinical way nor was it to imply that chimpanzees can be psychopaths. Rather, the goal was to evaluate the triarchic model from a comparative and evolutionary standpoint.

Results from this work indicate that the triarchic model of psychopathy can be operationalized effectively in chimpanzees, an animal species uniquely well-suited for neurobiological investigations of individual variation in broad, transdiagnostic biobehavioral traits (Latzman et al., 2016b). Such an approach is particularly opportune given the National Institute of Mental Health's (NIMH) research domain criteria (RDoC; Insel et al., 2010; Kozak and Cuthbert, 2016) initiative, encouraging investigators to consider psychopathology in terms of neurobehavioral dispositions. Indeed, as described previously (i.e., Latzman et al., 2016a) the dimensional constructs of the triarchic model can be viewed as trait-dispositional counterparts to RDoC constructs (Yancey et al., 2016).

It has long been theorized that psychopathy has heritable biological foundations (e.g., Karpman, 1946; Lykken, 1995), and an accumulating empirical literature supports the idea that genetic influences contribute to variance in psychopathic personality tendencies (Waldman and Rhee, 2006). Indeed, a replicable human literature has reported appreciable heritabilities for psychopathic tendencies (e.g., Blonigen et al., 2005, 2006; Viding et al., 2005; Brook et al., 2010; Bezdjian et al., 2011; Tuvblad et al., 2016). Taken together, the available research literature with humans clearly indicates an important contribution of genes to psychopathic tendencies.

Using the CHMP-Tri model, the finding of significant heritabilities of psychopathic tendencies has recently been confirmed in chimpanzees (i.e., Latzman et al., 2017). Consistent with findings in humans, results indicate significant genetic contributions to individual variability in psychopathic tendencies. Further, within the population of apes included in this study, some were raised by their biological mothers, whereas others were raised by humans for the first 3 years of life in a nursery. As described in more detail below, this quasi-experimental manipulation allowed for the explicit consideration of early social rearing experiences on estimates of heritability. When examined separately by early rearing background, consistent with previous findings for general personality dimensions (Latzman et al., 2015), the heritability of psychopathy dimensions varied by early social learning experiences: Whereas all three triarchic dimensions showed significant heritability among mother-reared participants, heritability was not evident for any dimension in the nursery-reared subsample (Latzman et al., 2017). All told, the existing literature, for both human and chimpanzee samples, provides clear evidence of a genetic contribution to psychopathy. To date, however, little is known concerning the specific genes associated with psychopathy.

Genes that underlie arginine-vasopressin (AVP), a phylogenetically conserved neuropeptide, constitute a particularly promising candidate, given the role of AVP in a range of complex social behaviors in both humans and nonhuman animals (Donaldson and Young, 2008). For example, within a sample of patients meeting diagnostic criteria for various personality disorders, cerebrospinal fluid AVP levels were found to correlate with a history of aggression (Coccaro et al., 1998). In addition to associations with direct measures of AVP, converging findings suggest an association between AVP receptor polymorphisms and a range of psychopathy-relevant social behaviors. For example, AVPR1A, the vasopressin V1A receptor gene, has been shown to be related to several social behaviors including pair bonding, territoriality, and aggression among voles, particularly males (e.g., Young and Wang, 2004; Hammock and Young, 2005, 2006). With regard to primates specifically, studies with humans have suggested an association between a similar repetitive element in the AVPR1A promoter and relevant social behaviors including altruism (Wassink et al., 2004) and pair bonding relationships (Walum et al., 2008). Further, with regard to personality traits in humans, AVPR1A promoter polymorphisms have been found to be associated with variability in a number of psychopathy-relevant traits (Patrick and Drislane, 2015) including increased novelty seeking, decreased harm avoidance (Walum et al., 2008) and increased reward dependence (Bachner-Melman et al., 2005).

Many of the association studies reviewed above have focused on the RS3 polymorphic repetitive element. In humans, the RS3 repeat region is housed within a larger, ~350 bp tandem duplicated region. The first of these duplicated regions, DupA, spans −3730 to −4074 bp relative to the transcription start site and contains a GT_20−26_ microsatellite, known as STR1. The second duplicated region, DupB, spans −3382 to −3729 bp and contains the complex microsatellite, RS3 ((CT)_6−14_(GT)_8−24_). In chimpanzees, approximately 65% of the AVPR1A alleles have a complete deletion of the DupB region, resulting in a 357 bp difference between the DupB^+^ and DupB^−^ alleles (Donaldson et al., 2008). As a result of the complete deletion of RS3 in some individuals, chimpanzees are a uniquely valuable animal species for assessing the potential effect of the RS3 polymorphic repetitive element on individual variability in neurobehavioral dispositional dimensions such as those aspects described in the triarchic model.

In chimpanzees, a small but generally consistent literature suggests an association between AVPR1A and personality. For example, Hopkins et al. (2012) found that traits of Dominance and Conscientiousness were associated with polymorphic variation in *AVPR1A*, particularly among males. Latzman et al. (2014) have reported similar findings with AVPR1A associated with factor-analytically derived Disinhibition and Dominance constructs. Similar to Hopkins et al., these associations were found to vary by participant sex. Staes et al. (2015) also reported converging results. Specifically, Staes et al. found sex-specific AVPR1A associations with behavioral observations of sociability. Further, findings of associations between AVPR1A and chimpanzee personality have also been reported by Wilson et al. (2017) who found AVPR1A to be associated with Conscientiousness and Extraversion, although not in sex-specific ways. All told, AVPR1A appears to be a promising candidate gene for research investigating the genetic basis of interpersonal dispositional traits, such as those described by the triarchic model of psychopathy.

In addition to unique AVPR1A polymorphisms, as described in more detail below, chimpanzees in the current study were raised in different rearing environments early in life. In both human and nonhuman animals, the genetic contribution to particular traits likely depends on distinct factors in the environment, resulting in the relevance of genes in some environments but not in others (Charmantier and Garant, 2005; Rutter et al., 2006). That is, the genetic contributions to various outcomes may differ depending on the environment. Across human (i.e., Moffitt et al., 2006), monkey (i.e., Suomi, 2011), and chimpanzee (i.e., Latzman et al., 2015, 2017) samples, research has suggested an interactive contribution of early adversity and genetic variation to a broad range of outcomes. Research in nonhuman animals (i.e., Charmantier and Garant, 2005), including chimpanzees (Latzman et al., 2015, 2017), has revealed similar variability in heritability estimates as a function of differences in early adversity. Indeed, as described above, recent CHMP-Tri findings suggest that the heritability of psychopathy dimensions varies by early social rearing experiences (Latzman et al., 2017). Given these existing lines of evidence, the role of early social rearing experiences is important to consider.

The overarching aims of the current study were to investigate the effects of DupB genotype on triarchic psychopathy dimensions and whether these effects appear sex-specific. Although no studies to date have investigated AVPR1A RS3 polymorphisms and psychopathy specifically, molecular genetic studies on the association between other specific candidate genes and psychopathy-related behaviors and traits suggest an important moderating role for early adversity in moderating associations (e.g., Capitanio et al., 2006; Kim-Cohen et al., 2006; Buckholtz and Meyer-Lindenberg, 2008; Karere et al., 2009). Similar findings have been shown among chimpanzees with regard to heritability estimates of psychopathy traits (Latzman et al., 2017). Specifically, whereas all psychopathy dimensions were found to be heritable among mother-reared apes, none of the heritability estimates were significant among nursery-reared apes. We thus decided to examine associations separately by early rearing experience.

Given previous findings of significant associations between AVPR1A and Conscientiousness, Dominance and Disinhibition (e.g., Hopkins et al., 2012; Latzman et al., 2014), we expected genotypic variability to relate as well to the CHMP-Tri dimensions, in distinct ways. Specifically, given previous chimpanzee findings with regard to Disinhibition (Latzman et al., 2014) and Conscientiousness (Hopkins et al., 2012), and clear links between triarchic disinhibition and each of these two constructs, we expected significant associations to emerge between AVPR1A genotype and CHMP-Tri Disinhibition, particularly for males. Specifically, we predicted that DupB^+/−^ males would show lower scores on this triarchic trait dimension. Further, given previous findings of lower levels of Dominance (Latzman et al., 2014), along with higher levels of anxiety-related behaviors (i.e., scratching, Latzman et al., 2016b; Mahovetz et al., 2016) for DupB^+/−^ males, we predicted that DupB^+/−^ males would score lower on CHMP-Tri Boldness, a triarchic disposititon associated with low threat-sensitivity. We did not advance any *a priori* hypotheses regarding CHMP-Tri Meanness. Whereas, previous human findings of an association between AVPR1A genotype and Dictator game performance (Knafo et al., 2008) are suggestive of an association for meanness, prior work with chimpanzees has reported no association between AVPR1A variability and scores on Agreeableness, the personality trait most strongly associated with triarchic meanness (Patrick and Drislane, 2015). Finally, given previous findings of a genetic foundation for a higher-order psychopathic personality dimension in both humans (e.g., Larsson et al., 2006) and chimpanzees (i.e., Latzman et al., 2017), we investigated the association between DupB genotype and a total CHMP-Tri score. Importantly, given previous findings of significant genetic contributions to CHMP-Tri scales among mother- but not nursery-reared chimpanzees, we expected to find significant AVPR1A effects only in the mother-reared sample.

## Materials and methods

### Participants

Chimpanzees were members of two genetically distinct colonies of apes housed at the Yerkes National Primate Research Center (YNPRC) in Atlanta, Georgia and at the National Center for Chimpanzee Care (NCCC) at The University of Texas MD Anderson Cancer Center in Bastrop, Texas. Participants for the current study included 82 adult and sub-adult chimpanzees at YNPRC, including 57 females and 25 males ranging in age from 9 to 53 years (*M*_*age*_ = 22.15, *SD* = 8.96) and 96 adult and sub-adult chimpanzees at NCCC, including 46 females and 50 males ranging in age from 8 to 41 years (*M*_*age*_ = 22.88, *SD* = 6.12). After removing 14 chimpanzees for whom AVPR1A data were not available (e.g., blood sample were not available, DNA yield was not sufficient) participants from both colonies were combined for analyses resulting in a final sample of 164 chimpanzees.

Early rearing experiences varied among individuals, with 119 being mother-reared and 59 human nursery-reared. Mother-reared chimpanzees remained under the care of their mothers for at least 2.5 years of life and were raised in “nuclear” family groups of chimpanzees, with group sizes ranging from 4 to 20 individuals. Nursery-reared chimpanzees were separated from their mothers within the first 30 days of life, due to unresponsive care, injury, or illness. These chimpanzees were placed in incubators, fed standard human infant formula, and cared for by humans until they could care adequately for themselves, at which time they were placed with other infants of the same age until they were 3 years old (Bard et al., 1992; Bard, 1994). At 3 years of age, the nursery-reared chimpanzees were integrated into larger social groups of adult and sub-adult chimpanzees.

It should be noted that all of the nursery-reared chimpanzees were raised in this manner to protect the infants' well-being. That is, the chimpanzees in this study were not nursery-reared by design, with the goal of subsequently determining the effects of early life experiences on development. The data for these subjects are therefore *ex post facto* and opportunistic; indeed, we capitalized on the fact that some of the chimpanzees received different rearing experiences in order to evaluate whether this might have long-term consequences on personality development. Importantly, as described previously (Bogart et al., 2014; Latzman et al., 2017), based on the composition of the rearing groups, potential rearing differences are not conflated with familial environment. That is, group membership reflects early experiences, rather than familial aggregation of group placement decisions. As reported previously by Latzman et al. (2017) with regard to relatedness within each rearing group, 52 different sires and 79 different dams contributed to the mother-reared group, and 34 different sires and 42 different dams contributed to the nursery-reared group. Further, as described in detail previously, the genetic diversity within each group was comparable suggesting that group membership reflects early experiences rather than familial aggregation of group placement decisions (see Latzman et al., 2017). The full pedigree structure for this sample has been described previously (see Hopkins et al., 2014b).

All aspects of the research complied with the American Psychological Association's Guidelines for Ethical Conduct in the Care and Use of Nonhuman Animals in Research (American Psychological Association, 2012), followed the Institute of Medicine (US) and National Research Council (US) Committee on the Use of Chimpanzees in Biomedical and Behavioral Research (2011) guidelines for research with chimpanzees, and was done with the approval of the Institutional Animal Care and Use Committees of the universities at which the research was conducted. All chimpanzees are housed in social groups ranging from 2 to 16 individuals in indoor-outdoor compounds, with free access to both portions of their enclosures 24 h a day. During the winter, the indoor facilities are heated, while air conditioning or fans and misters are provided in the hotter summer months. Lighting in the outdoor facility follows the typical seasonal cyclic change in sunrise and sunset. Standard tungsten lighting is provided in the indoor facility and the lights are on a 12 h on-off cycle. The chimpanzees are fed two to five times per day with a diet that consists of fruits, vegetables, and commercially produced primate chow. In addition, they receive a number of foraging and enrichment opportunities each day. Environmental enrichment, such as simulated tool use tasks or non-nutritive substrates, is provided to the chimpanzees on a daily basis. At no time are apes food- or water-deprived.

### Assessment of triarchic psychopathy dimensions

Chimpanzee Triarchic (CHMP-Tri) scales previously developed through a consensus-based approach (Latzman et al., 2016a) were used in the current study. Consistent with the triarchic model of psychopathy in humans, the three CHMP-Tri scales assess Boldness (6-items), Meanness (5-items), and Disinhibition (7-items). As described by Latzman et al. (2016a), chimpanzees were rated by colony-staff members; typically two to three independent raters, who had worked with the animals for an extended period of time and reported having “enough experience for an accurate rating” (Freeman et al., 2013, p. 1044), rated each chimpanzee. Items for each scale were rated using a 7-point Likert-type format, with response options ranging from 1 (*least descriptive of the chimpanzee*) to 7 (*most descriptive of the chimpanzee*). Internal consistencies (Cronbach's alpha) for the three scales have been shown to be acceptable, especially considering their brevity: 0.82 for Boldness, 0.77 for Disinhibition, and 0.67 for Meanness (Latzman et al., 2016a).

### DNA extraction, genotyping and analysis

As described previously (e.g., Donaldson et al., 2008), DNA samples were isolated from buccal swabs or blood samples using Puregene DNA purification system (Gentra, Minneapolis, MN, USA). Following extraction, stock DNA was separated into three aliquots: one for onsite storage at −80°C, one for offsite storage, and a working stock for genotyping. Samples were tracked via a secure Filemaker Pro 8 database that linked sample codes for each aliquot, demographics for each subject, DNA quantification and purity analysis results, and genotype data.

Each individual was genotyped for the *AVPR1A* DupA/B region using the primers and conditions reported in previous studies (e.g., Hopkins et al., 2012; Latzman et al., 2014). Briefly, we used forward primer 5′-GCATGGTAGCCTCTCTTTAAT and a reverse primer of 5′- CATACACATGGAAAGCACCTAA with an annealing temperature of 57°C for 30 cycles: 95°C, 5 min; 30 × (95°C, 30 s; 57°C, 30 s; 72°C, 3 min; 72°C, 10 min; 4°C, hold). Polymerase chain reaction (PCR) amplification was undertaken using the Epicentre Failsafe kit using premixH (Illumina Inc., Madison, WI, USA) according to the manufacturer's directions. Genotyping was performed in a volume of 20 μl containing 20 ng target genomic DNA. PCR products were resolved on a 2% agarose gel (SeaKem Agarose LE, Lonza, Basel, Switzerland) at 100 V for 45 min with a 100-bp DNA ladder (New England Biolabs, Ipswich, MA, USA) in tris-borate-EDTA (TBE). The DupB-containing allele resulted in a band of ~900 bp, while the DupB minus allele was ~570 bp long, and genotypes were visually assigned (Donaldson et al., 2008). All genotypes were run in duplicate with gel analysis and were checked before the data set was finalized. Forty-three males and 64 females were homozygous for the short allele (DupB^−/−^) and 26 males and 31 females had the long allele (DupB^+/−^), yielding overall genotype frequencies of 65.2 and 34.8%. These frequencies are consistent with those previously identified in wild-caught chimpanzees (Donaldson et al., 2008). Further, as reported in previous studies, the AVPR1A genotype distribution in these two colonies of apes does not deviate from Hardy-Weinberg equilibrium (Hopkins et al., 2014a).

### Data analysis

To examine associations between CHMP-Tri dimensions and AVPR1A DupB genotype, we used multivariate analysis of variance (MANOVA). Specifically, we used CHMP-Tri scores as the dependent variables and sex as a fixed factor, and, given the age range of the sample, we included age as a covariate. The potential moderating role of sex was examined for a number of reasons. In addition to vasopressin systems in the brain being (1) found to be sexually dimorphic and (2) thought to regulate social behaviors in sex-specific ways (De Vries et al., 1981), investigations in chimpanzees of associations between AVPR1A and general dispositional traits have resulted in sexually-dimorphic results (e.g., Hopkins et al., 2012; Latzman et al., 2014, 2016b). Given prior work with chimpanzees demonstrating important moderating impacts of early rearing experience on the heritability of personality broadly (i.e., Latzman et al., 2015), and psychopathy more specifically (i.e., Latzman et al., 2017), analyses were conducted separately for mother- and nursery-reared chimpanzees.

To confirm that associations between AVPR1A DupB genotype and CHMP-Tri scores were a direct reflection of AVPR1A rather than a result of all shared genes, a series of *post-hoc* analyses were performed in the Sequential Oligogenic Linkage Analysis Routines (SOLAR; Almasy and Blangero, 1998) program. Specifically, as described in detail previously (see Fears et al., 2009, 2011), and consistent with recent quantitative genetic work on CHMP-Tri scales (Latzman et al., 2017), SOLAR uses a variance components approach that relies on maximum likelihood estimation to compute a polygenic variance term for a dependent measure of interest (i.e., CHMP-Tri scores) when considering the entire pedigree. To determine the contribution of AVPR1A DupB genotype explicitly, we included AVPR1A and AVPR1A^*^sex as covariates in the polygenic models for each CHMP-Tri score found associated with AVPR1A in our MANOVA analyses. The contribution of genotype and genotype^*^sex to the explanation of the CHMP-Tri scores independent of genetic relatedness was evaluated by testing the statistical significance of their associations within the full model.

## Results

### AVPR1A variation and Chmp-Tri psychopathy dimensions

**Figure 1:**
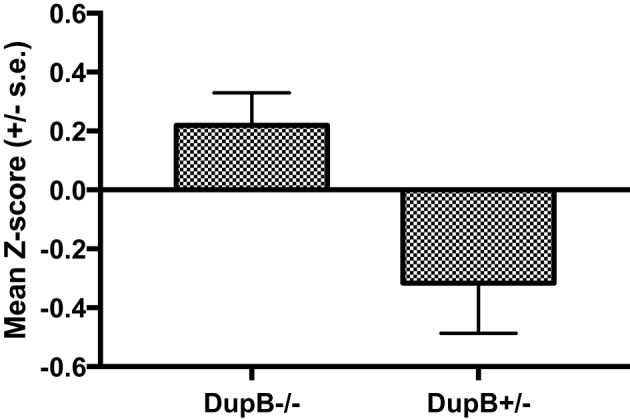
Mean CHMP-Tri Disinhibition scores (± SE) for mother-reared chimpanzees with DupB^+/−^ and DupB^+/−^ GENOTYPES. *n* = 77 for DupB^−/−^. *n* = 34 for DupB^+/−^.

Within the mother-reared sample, a significant main effect was found for AVPR1A[*F*_(3, 104)_ = 8.78, *p* < 0.001, η_*p*_^2^ = 0.10] in the prediction of CHMP-Tri scores. Subsequent univariate *F*-tests revealed a significant main effect association for DupB genotype with CHMP-Tri Disinhibition [*F*_(1, 106)_ = 6.66, *p* = 0.01, η_*p*_^2^ = 0.06]. Mean standardized CHMP-Tri Disinhibition scores in DupB^−/−^ and DupB^+/−^ are shown in Figure 1. Specifically, across sexes, Dup^+/−^ apes scored significantly higher in disinhibitory tendencies than DupB^−/−^ apes. By contrast, no direct effects between the DupB genotype and either CHMP-Tri Boldness [*F*_(1, 106)_ = 2.75, *p* > 0.10, η_*p*_^2^ = 0.03] or Meanness emerged [*F*_(1, 106)_ = 1.28, *p* > 0.25, η_*p*_^2^ = 0.01].

**Figure 2:**
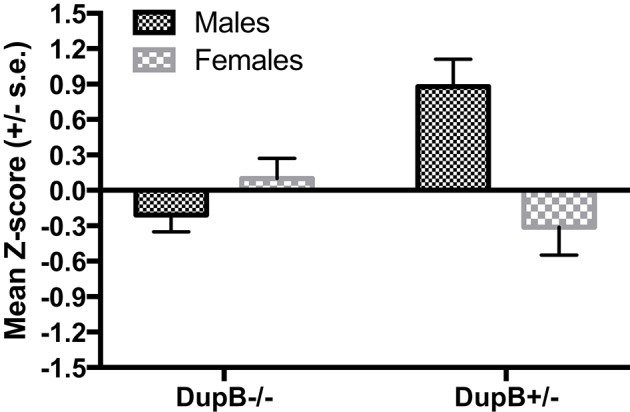
Mean CHMP-Tri boldness scores (± SE) for mother-reared males and females with DupB^+/−^ and DupB^−^ genotypes. *n* = 29 DupB^−/−^ males and 48 DupB^−/−^ females. *n* = 16 DupB^+/−^ males and 18 DupB^+/−^ females.

However, a significant two-way interaction between AVPR1A and sex [*F*_(3, 104)_ = 4.87, *p* < 0.01, η_*p*_^2^ = 0.12] was found in predicting CHMP-Tri scores as a whole. Subsequent univariate *F*-testsrevealed that this omnibus effect was attributable mainly to the predictive effect of the AVPR1A^*^sex interaction for Boldness [*F*_(1, 106)_ = 14.70, *p* < 0.001, η_*p*_^2^ = 0.12]. Mean standardized CHMP-Tri Boldness scores in DupB^−/−^ and DupB^+/−^ males and females are shown in Figure 2. Whereas, female Dup^−/+^ apes evidenced higher scores on this trait dimensions, male Dup^−^/+ apes showed lower scores on CHMP-Tri Boldness. No significant effects for the AVPR1A^*^sex interaction term were found in predicting CHMP-Tri Meanness [*F*_(1, 106)_ = 2.71, *p* > 0.10, η_*p*_^2^ = 0.03] or Disinhibition [*F*_(1, 106)_ = 0.51, *p* > 0.45, η_*p*_^2^ = 0.01].

Within the nursery-reared sample, no significant main effects [*F*_(3, 46)_ = 0.06, *p* > 0.95, η_*p*_^2^ = 0.004] or interactions [*F*_(3, 46)_ = 0.16, *p* > 0.90, η_*p*_^2^ = 0.01] were found. No predictive effects were found for any individual CHMP-Tri scale (all *F*s < 0.20, *p*s > 0.65).

### AVPR1A variation and total CHMP-Tri psychopathy

**Figure 3:**
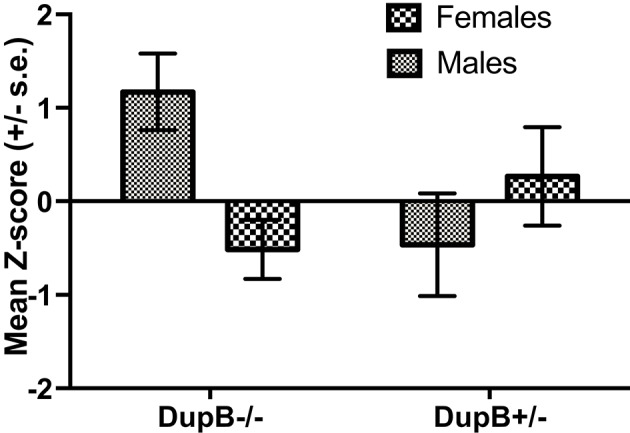
Mean CHMP-Tri total scores (± SE) for mother-reared males and females with DupB^+/−^ and DupB^+/−^ genotypes. *n* = 29 DupB^−/−^ males and 48 DupB^−/−^ females. *n* = 16 DupB^+/−^ males and 18 DupB^+/−^ females.

Finally, the association between DupB genotype and total CHMP-Tri psychopathy was investigated. Within the mother-reared sample, whereas no significant main effect for AVPR1A emerged [*F*_(1, 106)_ = 0.83, *p* > 0.35, η_*p*_^2^ = 0.01], a significant two-way interaction between AVPR1A genotype and sex [*F*_(1, 106)_ = 7.13, *p* < 0.01, η_*p*_^2^ = 0.06] was found in the prediction of total CHMP-Tri scores. Mean standardized CHMP-Tri total scores in DupB^−/−^ and DupB^+/−^ are shown in Figure 3. Whereas, female DupB^−/+^ apes evidenced lower scores, male Dup^−/+^ apes showed higher scores on CHMP-Tri total score; the opposite was true for DupB^−/−^ apes. Within the nursery-reared sample, no significant main effects [*F*_(1, 53)_ = 0.03, *p* > 0.85, η_*p*_^2^ = 0.001] or interactions [*F*_(1, 53)_ = 0.03, *p* > 0.85, η_*p*_^2^ = 0.001] were found.

### Contribution of AVPR1A and AVPR1A^*^Sex in polygenic models

As described above, to confirm the robustness of our MANOVA findings within the mother-reared group, tests of AVPR1A and AVPR1A^*^sex as potential covariates within full polygenic models were next run. Consistent with MANOVA findings, these *post-hoc* analyses suggested a significant specific effect for AVPR1A DupB genotype for CHMP-Tri Disinhibition explaining 8.56% of the proportion of variance (*p* = 0.01). Further, also consistent with MANOVA findings described above, AVPR1A^*^sex evidenced a significant effect for both CHMP-Tri Boldness explaining 8.32% of the variance (*p* = 0.0009) and CHMP-Tri Total score explaining 5.86% of the variance (*p* = 0.005).

## Discussion

The current study represents the first investigation to date of the association between variation in AVPR1A, a gene that underlies AVP, and psychopathy tendencies as a function of differences in early rearing experiences. Consistent with expectations, AVPR1A DupB genotype was found to explain variability in psychopathy dimensions uniquely and in sex-specific ways. Results underscore the translational value of a nonhuman primate model for investigating psychopathy dimensions and provide strong support for the notion of triarchic psychopathy dimensions as biologically-based and evolutionarily derived (Patrick et al., 2009; Latzman et al., 2016a).

### AVPR1A and triarchic psychopathy

Consistent with previous findings in both humans and chimpanzees with regard to psychopathy-related behaviors and traits, results suggest a potentially important etiological pathway from AVP to triarchic psychopathy, at least for mother-reared apes. Specifically, in line with previous findings of higher scores on the personality traits of Disinhibition/low Conscientiousness (Hopkins et al., 2012; Latzman et al., 2014), traits linked to triarchic disinhibition (Patrick and Drislane, 2015), DupB^+/−^ apes evidenced lower scores on CHMP-Tri Disinhibition in the current sample. In contrast to previous findings of this association being male-specific, however, this association did not vary by sex in the current study. Contrary to both expectations and previous findings for related traits and behaviors, DupB^+/−^ was found to be associated with higher CHMP-Tri Boldness scores for males. This is surprising for a number of reasons, including previous findings of higher levels of anxiety-related behaviors (i.e., scratching, Latzman et al., 2016b; Mahovetz et al., 2016) for DupB^+/−^ males and the contention that triarchic boldness reflects the phenotypic expression of low threat-sensitivity. It will thus be important for future research to more explicitly investigate this finding. Finally, total CHMP-Tri psychopathy was found to associate with AVPR1A in sex-specific ways. Importantly, in a series of *post-hoc* analyses, the robustness of findings and their specificity to AVPR1A rather than all shared genes, were confirmed through a series of polygenic models in which genetic relatedness among apes were considered. All told, although not entirely consistent with expectations, the current findings in the mother-reared sample suggest an important role for AVP on variation in psychopathy dimensions.

In direct contrast to findings among mother-reared chimpanzees, and consistent with recent biometric results (Latzman et al., 2017), none of the psychopathy dimensions were found to associate with the AVPR1A RS3 polymorphism in the nursery-reared sample, providing evidence of different etiologies as a function of rearing. These findings are not only consistent with biometric results in chimpanzees, but also with accumulating evidence in the human (e.g. Moffitt et al., 2006; Rutter et al., 2006) and nonhuman animal literatures (e.g., Charmantier and Garant, 2005; Karere et al., 2009) for variations in the effects of genes as a function of environmental context. Importantly, whereas in humans it is quite difficult to disentangle etiological influences due to confounding of environmental and genetic influences, findings for our chimpanzee sample are less likely to reflect this confound. Indeed, as described previously (i.e., Bogart et al., 2014; Latzman et al., 2017), although offspring in each of the two early rearing groups were not entirely heterogeneous, the degree of genetic diversity was comparable between them.

### Triarchic model, AVP, and the NIMH research domain criteria (RDoC)

A notable feature of the dispositional constructs of the triarchic model is that they are framed explicitly in neurobiological terms (Patrick et al., 2009; Patrick and Drislane, 2015). As mentioned at the outset, an analysis of psychopathy in terms of neurobehavioral dispositions is apt, given the NIMH's RDoC initiative (Insel et al., 2010; Kozak and Cuthbert, 2016), which endeavors to explicate the neurobiological bases of mental illness and reframe conceptions of psychopathology around constructs with specific brain referents. The RDoC research framework specifies biobehavioral constructs, grouped within major domains of functioning, as explanatory referents for understanding clinical problems—and encourages investigation of these constructs using measures from multiple assessment domains (“units of analysis”). Clear counterparts to the triarchic model dimensions exist within the RDoC framework. Indeed, Disinhibition fits within the RDoC construct of “response inhibition” within the Cognitive Systems domain; boldness fits within the construct of “acute threat” in the Negative Valence Systems domain; and meanness fits within the construct of “affiliation and attachment” in the Social Systems domain. The dimensional constructs of the triarchic model can thus be viewed as trait-dispositional counterparts to these RDoC constructs (Yancey et al., 2016).

The direct relevance of the current research to the RDoC initiative is further bolstered by the consideration of relations between these three biobehavioral phenotypes and AVPR1A. Indeed, the vasopressin system is explicitly referred to as a suggested unit of analysis across a variety of RDoC domains and constructs, including within the Negative Valence System, Social Processes, and Arousal and Regulatory Systems Domains. For example, vasopressin is thought to relate to the Acute Threat (“Fear”) construct within the Negative Valence System, a construct with direct links to boldness in the triarchic model.

Although the current study focused specifically on psychopathy subdimensions described within the triarchic model and associations with the AVPR1A RS3 polymorphism, taken together with a growing body of research (i.e., Latzman et al., 2016b), results from the current study provide clear support for primate-translational operationalizations of specific constructs within the RDoC framework. Despite recent decisions by the National Institutes of Health (National Institutes of Health, 2011) to scale back research of some types involving captive chimpanzees, work undertaken for the current study fits clearly within the ethical framework of scientifically justifiable research with chimpanzees as outlined by the Institute of Medicine (Institute of Medicine (US) and National Research Council (US) Committee on the Use of Chimpanzees in Biomedical and Behavioral Research, 2011). In conjunction with work being conducted on other RDoC-relevant lines (e.g., Hopkins et al., 2014a; Latzman et al., 2016b), the current work highlights the importance of including a chimpanzee comparative-translational component in the NIMH RDoC research program. Along with findings from human studies, work of this kind can provide exceptionally valuable insights into core biobehavioral processes relevant to psychological illness and health (Latzman and Hopkins, 2016).

### Limitations

The current study is not without limitations. First, the sample size, particularly in the case of the nursery-reared subgroup, was relatively modest. Additional research is thus needed to replicate the current findings and establish more stable estimates for contributions of AVP to psychopathy. Nonetheless, it is important to note that although potential concerns regarding sufficient power to detect effects within the nursery-reared sample are appropriate, effect sizes for these associations approached zero (e.g., η_*p*_^2^ ≤ 0.01). Additionally, while widely-used in both the human and nonhuman primate literatures, our use of scores on the CHMP-Tri scales, derived from caretaker ratings of a set of adjective descriptors with accompanying narrative definitions, is only one of a number of potential approaches to assessing the dimensions described within the triarchic model. Indeed, multi-domain operationalizations of triarchic dimensions are possible through the use of composite psychoneurometric indices of the various dimensions (e.g., Patrick et al., 2013; Yancey et al., 2016). It will be important for future research to replicate the current findings considering various domains of measurement.

Additionally, there have been a number of replicability concerns raised with regard to candidate gene studies (e.g., Munafo, 2009). Although AVPR1A has repeatedly been found to underlie variation in AVP, the current study considered a single polymorphism and did not directly assess circulating levels of AVP. Further, we are not able to determine whether the differences that emerged are due directly to gene expression caused by the presence or absence of the DupB region. Nonetheless, as described earlier, across nonhuman animal studies, AVPR1A has emerged as a reliable correlate of a variety of social behaviors and traits. One important strength of animal studies is that, as compared to humans, nonhuman animal participants are raised in homogeneous, controlled environments; indeed, as described above, chimpanzee participants in the current study were raised in a common, controlled environment. Nonetheless, it will clearly be important for future studies to replicate our findings and also include more direct measures of AVP to confirm that our findings are a result of AVPR1A expression or some other potential pathway. Further, it will be important for future studies to examine additional related neuropeptides known to be associated with social behavior, such as oxytocin and associated genes.

Finally, it is important to note that chimpanzees encounter a variety of potentially impactful early experiences, whether raised by their biological mothers or in human-managed nursery settings. Given this, as noted previously (i.e., Latzman et al., 2015), our classification of participants into subgroups based on the ostensibly topographical manner in which they were raised likely obscures important variability within each group. Notably, however, our approach of grouping chimpanzee participants in this manner likely resulted in a more conservative indication of the role of early social rearing experiences, potentially enhancing confidence in conclusions advanced from current findings. Relatedly, the nursery-rearing experience of apes is not directly parallel to experiences of early adversity in humans. As described previously (e.g., Latzman et al., 2017), nursery-reared apes are removed from their mothers as a result of caregiving needs (e.g., inadequate maternal care, injury, illness) and subsequently placed in an adequate (i.e., less adverse) condition. Nonetheless, whereas this is not the best parallel to experiences of physical adversity, this experience is likely more similar to human experiences of social adversity. Indeed, maternal deprivation at an early age is associated with a number of dysfunctional behaviors in humans (Gunnar and Quevedo, 2007). Thus, although likely not completely analogous to the early adversity encountered by many humans, it is clear that early maternal separation results in a number of behavioral sequelae indicative of adversity.

## Conclusion

Using a powerful and unparalleled animal model, the current study points to contributions of AVP influences to psychopathic tendencies, with an important role for a specific environmental factor—early rearing experience—in affecting this contribution. Taken together, results suggest an important contributory influence of neuropeptide variation in the explanation of the development of psychopathy. Results of the current study further provide additional compelling evidence that psychopathic tendencies are rooted in basic, evolutionarily-meaningful dispositions (Fowles and Dindo, 2006; Patrick et al., 2009; Skeem et al., 2011; Patrick and Drislane, 2015; Latzman et al., 2016a, 2017), and provide support for a primate-translational operationalization of key neurobehavioral constructs relevant both to psychopathy and to broader forms of psychopathology. As such, the current work highlights the value of a chimpanzee comparative-translational component to the NIMH RDoC research framework (Latzman and Hopkins, 2016).

## Author contributions

RL and WH conceived of the study. WH and SS oversaw data collection. RL performed all analyses and drafted the paper. WH and SS provided critical revisions.

### Conflict of interest statement

The authors declare that the research was conducted in the absence of any commercial or financial relationships that could be construed as a potential conflict of interest.
